# Quantification of Facial Traits

**DOI:** 10.3389/fgene.2019.00397

**Published:** 2019-05-24

**Authors:** Stefan Böhringer, Markus A. de Jong

**Affiliations:** Department of Biomedical Data Sciences, Leiden University Medical Center, Leiden, Netherlands

**Keywords:** face, quantification, registration, 3D surface, photogrammetry, reliability, dimension reduction, landmark

## Abstract

Measuring facial traits by quantitative means is a prerequisite to investigate epidemiological, clinical, and forensic questions. This measurement process has received intense attention in recent years. We divided this process into the registration of the face, landmarking, morphometric quantification, and dimension reduction. Face registration is the process of standardizing pose and landmarking annotates positions in the face with anatomic description or mathematically defined properties (pseudolandmarks). Morphometric quantification computes pre-specified transformations such as distances. **Landmarking:** We review face registration methods which are required by some landmarking methods. Although similar, face registration and landmarking are distinct problems. The registration phase can be seen as a pre-processing step and can be combined independently with a landmarking solution. Existing approaches for landmarking differ in their data requirements, modeling approach, and training complexity. In this review, we focus on 3D surface data as captured by commercial surface scanners but also cover methods for 2D facial pictures, when methodology overlaps. We discuss the broad categories of active shape models, template based approaches, recent deep-learning algorithms, and variations thereof such as hybrid algorithms. The type of algorithm chosen depends on the availability of pre-trained models for the data at hand, availability of an appropriate landmark set, accuracy characteristics, and training complexity. **Quantification:** Landmarking of anatomical landmarks is usually augmented by pseudo-landmarks, i.e., indirectly defined landmarks that densely cover the scan surface. Such a rich data set is not amenable to direct analysis but is reduced in dimensionality for downstream analysis. We review classic dimension reduction techniques used for facial data and face specific measures, such as geometric measurements and manifold learning. Finally, we review symmetry registration and discuss reliability.

## 1. Introduction

The face plays an important role in human interaction and is scientifically researched with respect to many disciplines including genetic control (Boehringer et al., 2011b; Liu et al., 2012; Paternoster et al., 2012; Adhikari et al., 2016; Cole et al., 2016; Shaffer et al., 2016; Lee et al., 2017; Claes et al., 2018), psycho-social impact (Scheib et al., 1999; Leyvand et al., 2008), archaeology (Stenton et al., 2016), forensic reconstruction (Short et al., 2014), relation with medical conditions (Hammond et al., 2005; Boehringer et al., 2006, 2011a; Vollmar et al., 2008; Wilamowska et al., 2012), and facial identification (Wiskott and Von Der Malsburg, 1996; Schroff et al., 2015; Sun et al., 2015).

Facial identification or face recognition is the process of determining whether a face as represented by a single or a few images is present on a given target image. We do not cover face recognition in this review and only provide a brief discussion. In this review, we cover automatic methods that can derive quantitative values, such as distances, from given facial raw data as represented by 2D photographs or 3D surface scans. Such quantities are of interest in genome wide association studies (GWASs), syndrome classification, and other prediction settings.

Early applications of facial genetics focused on Mendelian patterns (Lebow and Sawin, 1941; Vandenberg and Strandskov, 1964) of rough facial measurements such as length or heritability of measurements derived from lateral skull radiographs(Hunter et al., 1970; Nakata et al., 1976). A first statistical quantification proposed a harmonic analysis of cephalometric data by considering the outer rim of the skull (Lu, 1965). In the following papers, quantitative facial analyses were applied to determine syndrome diagnoses (Hammond et al., 2005; Boehringer et al., 2006, 2011a; Vollmar et al., 2008; Wilamowska et al., 2012), and to identify faces (Wiskott and Von Der Malsburg, 1996; Schroff et al., 2015; Sun et al., 2015). Many syndromes can be well-classified due to distinct facial characteristics between the affected group and healthy controls. The syndrome groups were relatively small in number (100's of individuals) allowing manual annotation of faces. The introduction of GWASs (Liu et al., 2012; Paternoster et al., 2012; Adhikari et al., 2016; Cole et al., 2016; Shaffer et al., 2016; Lee et al., 2017; Claes et al., 2018) required large cohort sizes to attain sufficient statistical power. While a limited number of landmarks have been placed manually (Paternoster et al., 2012), (semi)automatic procedures have also been utilized (Liu et al., 2012; Adhikari et al., 2016; Lee et al., 2017; Claes et al., 2018). As cohort sizes increase further, reliable automatic quantification becomes more important.

In this review, facial quantification is divided into three steps: pre-processing (face registration), landmarking (facial alignment), and deriving outcome measures based on these landmarks (morphometric quantification, dimension reduction). While, in principle, landmarks are not necessary to derive quantities from faces, they help in interpreting results and to remove important sources of variation from the data. We define face registration as the process of bringing a face into a well-defined pose. By facial alignment we mean that some or all points of a given face can be transformed to points on another face while retaining their meaning in terms of landmarks, thereby establishing correspondence between landmarks. This is intuitively obvious for anatomic landmarks (Swennen, 2006) but can be extended to a full facial surface through dense surface models or pseudo-landmarks. Finally, aligned landmark data is rarely analyzed directly. Rather they are processed further using either pre-specified transformations or dimension reduction techniques. Important examples are morphometric quantities such as distances. Other transformations are derived from the data, which we call global quantification because they operate on the full data set. Principal component analysis (PCA) is an example of such a technique. These quantities are then used in ensuing analyses such as classification, heritability estimation, or genetic association.

As a final aspect, symmetry is quickly discussed as a face specific application and some open problems are mentioned.

## 2. Face Registration

Face registration is the process of aligning the face into a standard pose. In general, the definition of the standard pose is method specific. For example, nose, eyes, and mouth all offer possibilities for such definitions by aligning them to pre-specified locations. In principle, all landmarking methods can be trained to use non-registered faces, yet almost all methods are likely to profit when faces are pre-registered. The degree by which landmarking efforts depend on face registration certainly differs between methods. For example, template based methods use average image patches derived from training samples to represent landmarks (described in more detail in section 3.1), which can be used to locate landmarks in new data. Arguably, template based methods depend more on face registration than some other landmarks registration methods as the templates have been derived under a specific pose in the training data.

Viola and Jones (2004) proposed a 2D algorithm that turned out to be very robust for defining regions containing faces under greater variation of facial poses. This is important as in 2D, it is difficult to correct pose, as such a correction is a three-dimensional rotation which requires to estimate depth information first.

In 3D, available registration methods are heuristic, i.e., based on *ad-hoc* rules. They focus on characteristics of facial 3D models that are pre-selected based on plausible geometric assumptions. For example, a cylinder fitting approach with a 2D symmetry plane detection that iteratively converges toward symmetry between the left and right hand sides of the face was proposed (Spreeuwers, 2011). Other popular registration methods are based on curvatures. Using mean curvatures and relative positions to locate the nose tip and both inner eye corners (Sun and Yin, 2008) has been a successful approach.

After face registration, the surface models can be brought into the standard pose and can be automatically landmarked without taking into account pose. This contrasts with the application of deep neural networks (DNN). In such models, pose is learned as part of the landmarking model and helps to improve landmarking by generating features that represent pose (Bulat and Tzimiropoulos, 2017) (section 3.3).

## 3. Landmarking

Landmarking is the process of searching for locations on a given representation of a face corresponding to locations on a second such representation, or alternatively to those on an idealized face. Anatomical landmarks are defined anatomically (Swennen, 2006), and pseudo-landmarks are locations with a mathematical definition relative to anatomical landmarks. Often pseudo-landmarks are defined by the movement required to align anatomical landmarks that involve a corresponding movement of pseudo-landmarks in between, which is discussed in section 5.4. Finding corresponding landmarks allows for a statistical analysis with respect to genetics or other traits.

Most importantly, the availability of anatomical landmarks is the pre-requisite for further landmarking efforts. In many studies, anatomical landmarks are placed manually (Boehringer et al., 2011b; Paternoster et al., 2012), with a minimum of two required to define pseudo-landmarks. In the following, we discuss automated landmarking procedures for anatomical landmarks. Reliability of manual landmarking is discussed in section 6 and pseudo-landmarks in section 5.4.

### 3.1. Template Based Methods

#### 3.1.1. 2D Methods

A template for a landmark is defined as an image patch around a landmark that later can be used for comparison in a target image. Templates can be averages of image patches across a set of training images or the full set of image patches as extracted from training images.

Early attempts used image patches directly without transformations, and correlations of a template with a patch of a target image was used to locate either the total face or sub-features like eyes, nose, or mouth (Samal and Iyengar, 1992). Many modifications were developed such as weighted correlation (Kalina, 2010) or making templates adaptive to varying conditions as reviewed elsewhere (Yang et al., 2002). The review discusses methods for templates that can adapt to different lighting and pose conditions using parameters (parametrized templates) and also broadly discusses face identification methods.

Apart from using raw image intensities, templates can be represented after being transformed. In many cases, a wavelet decomposition is applied to the image and the resulting representation is stored instead (wavelet coefficients). Roughly speaking, in a simple case, a square image is subdivided into four smaller squares and a difference between average pixel intensities of these sub-squares is stored (wavelet coefficient). The wavelet itself is a function that allows and defines this computation. This process is repeated for the four squares, implying wavelet size shrinks by a factor of two with every step. Wavelet coefficients again represent how different the smaller sub-squares are within their embedding square. This process can be repeated until the squares contain only a single pixel or after a number of predefined steps. The original image can be reconstructed from this representation. Intuitively, the global average of pixel intensities and also differences between sub-squares for the first steps are usually uninformative for face or landmark detection. Only when the wavelet size is close to a patch size that spans useful facial features (e.g., a patch spanning the edge of the nose but not the whole nose) they become useful for landmarking purposes. In this sense, a wavelet decomposition is more informative than the raw image as information is represented on different spatial scales. For example, smaller wavelets can be used to represent sharp edges whereas larger wavelets coefficients correlate with softer gradients. In this way, relevant information is encoded in fewer numbers as compared to the pixel values of the raw image patch. In computer vision applications, this process usually does not start with pre-defined patches (squares) but is centered around points of interest (e.g., potential landmarks). The same properties are used in image compression such as the JPEG standard (Wallace, 1991), when coefficients represent different levels of detail and can be omitted when they only minimally affect image appearance. The ability of wavelet coefficients to sparsely describe image patches makes them attractive for computer vision applications. Details describing wavelet decompositions are given elsewhere (Gomes and Velho, 2015).

Haar-wavelets (Papageorgiou et al., 1998), which are computed as described in the previous paragraph (with details omitted), were introduced for the purpose of general object detection. For a particular type of object, a supervised learning step is used to identify which Wavelet coefficients are needed for detection. The original paper (Papageorgiou et al., 1998) already considers face detection, and was extended in several ways to allow for scale invariant detection and to improve computational efficiency (Viola and Jones, 2004). This became the Viola-Jones algorithm mentioned previously. The algorithm is implemented in the OpenCV library (Bradski, 2000) and has become an important tool for real-time applications and as a pre-processing step in other algorithms.

Gabor-Wavelet are a smooth variant of Haar-wavelets that also allow for overlap between wavelets and are used by the so-called elastic bunchgraph method (Wiskott and Von Der Malsburg, 1996; Wiskott et al., 1997). An additional modification was that templates are not averaged across training samples but are stored as a collection in the so-called *bunch graph*. When searching for a maximum correlation match in a target image, the whole set of templates is iterated and the maximum across all training examples is chosen, which allows to represent heterogeneity across samples. Also template based methods usually take into account some geometric information. Usually, the relative landmark positions are not allowed to deviate strongly from an average graph as expressed by a distance or deformation energy (Wiskott et al., 1997; Viola and Jones, 2004). For this reason, template based methods handle texture information very flexibly but are less flexible geometrically as compared to, for example, active shape models described in the next section. The methods described above have been shown in the corresponding papers to perform well when few training images are used. This was demonstrated by either showing qualitative examples (Kalina, 2010), face recognition rates (Wiskott et al., 1997), or by reporting landmarking accuracy (de Jong et al., 2018b). For many landmarks, 1–2 mm of accuracy can be achieved as compared to human raters.

#### 3.1.2. 3D Methods

Template based 2D based methods can be applied to 3D surface scans by first projecting scans to 2D. In an extension, the height of projected points can be stored in an elevation map as well. In this case, full information is retained as the projection can be reverted by using the elevation map. Using both sources of information can improve landmarking accuracy (de Jong et al., 2016). It is also possible to combine several landmarking algorithms into a combined method (ensemble) (de Jong et al., 2018b) which can increase flexibility (de Jong et al., 2018a).

### 3.2. Active Shape Models

**Figure 1:**
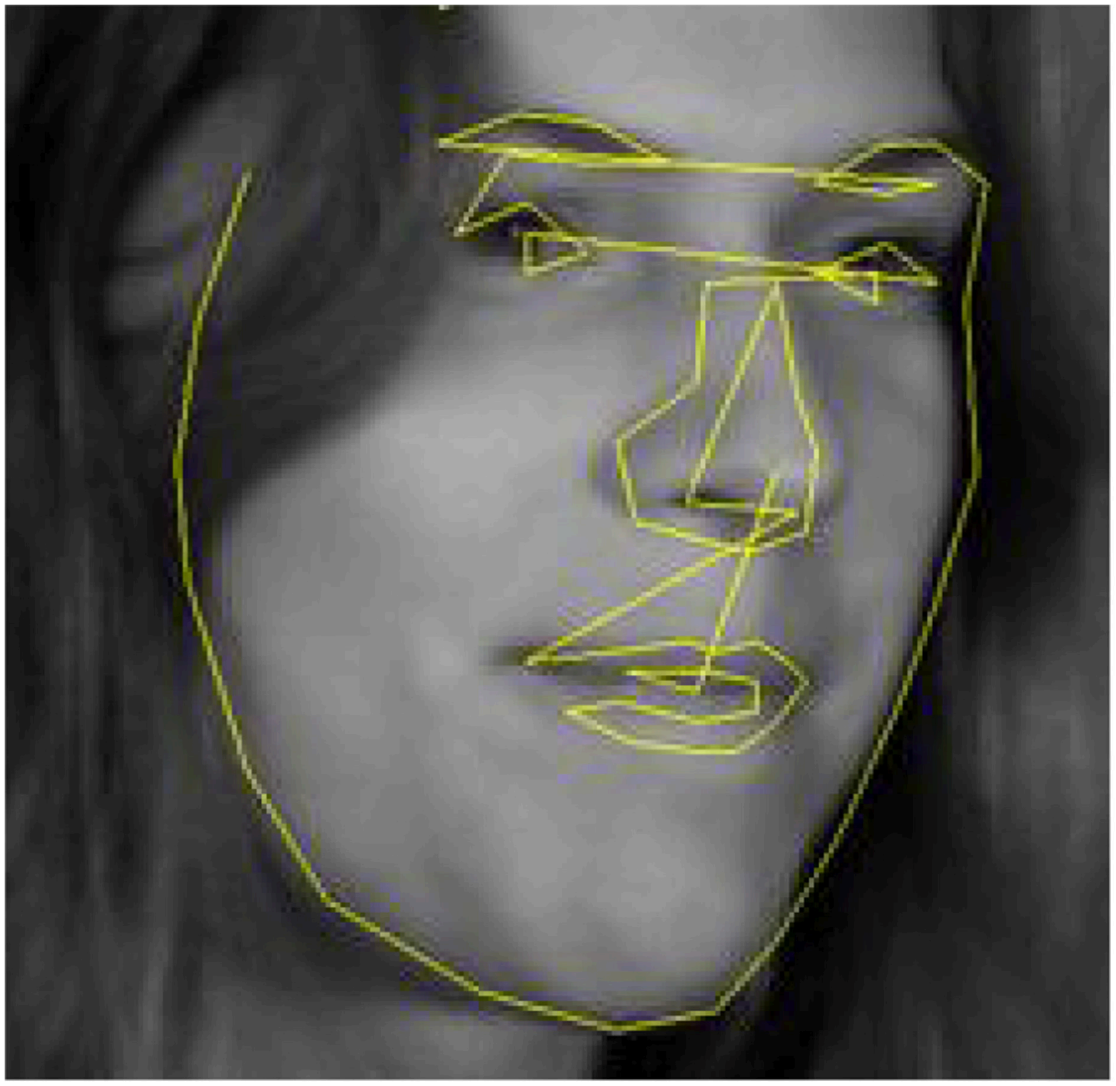
ASM alignment of a connected graph to a face. Source: wikipedia.

Active contour models (ACMs) place a graph on an image and try to align it with existing edges. The graph that requires minimal movement and deformation while matching image edges is the solution of the alignment process. An example is shown in Figure 1. More formally, the solution is a trade-off between fit to edges (measured by a distance) and the needed deformation (measured by an energy) (Kass et al., 1988). Active shape models (ASMs) use information from a training sample to improve the procedure. The sample is used to define deformations of the initial graph that have actually been seen in training samples (possible shapes). For example, in the case of faces, the ratio of height to width is constrained and ASMs would not consider a deformed graph violating this constraint, i.e., only the set of possible shapes is used in the search. The initial work assumed that all landmarks can be connected by edges that correspond to edges in the image (Cootes et al., 1995). Procrustes alignment is performed on the landmarks (see section 5) and a principal component analysis (PCA; see section 7) is performed on the aligned landmarks to define shapes. This is called the point distribution model (PDM). The PCA can be used to define shapes that are likely to be seen in new data by assuming that the training sample represents the true distribution. The landmarking process is iterative in trying to move landmarks toward edges in the image while constraining the combined set of proposed movements for all landmarks to a shape that is “likely enough” under the PDM.

When a proposed shape is too unlikely, it is moved back to the closest point in the acceptable region. The ASM approach has been extended to 3D, in the volumetric sense, i.e., voxel (Hill et al., 1993), and to 3D data as represented by multiple views (Milborrow et al., 2013; Montúfar et al., 2018).

One of the early applications of ASMs was landmarking of faces (Lanitis et al., 1997). The approach has been adapted to improve performance on faces where edges are not always appropriate to describe landmarks. The edge search has been modified to include the 2D profile around the edge (Milborrow and Nicolls, 2008) and outright templates (Milborrow and Nicolls, 2014) which have been chosen to be scale and rotation invariant (Lowe, 2004).

When ASMs are implemented for 2D images, a projection step from 3D to 2D can be used to apply these models on surface data. This has recently been performed when STASM (Zhou et al., 2009; Milborrow and Nicolls, 2014), an implementation of an ASM, has been compared to a template based approach (de Jong et al., 2016). In this comparison, STASM showed a landmarking accuracy of 1–2 mm. In contrast to ASMs, Active appearance models (AAMs) is not only based on edge information but also takes into account gray scale information of the full image (Edwards et al., 1998).

An attractive feature of ASMs is that facial expression is implicitly captured in the PDM. Based on a classification of facial expression by human raters, facial expression can be predicted from images. As facial expression has not yet been analyzed genetically, we only suggest two reviews (Fasel and Luettin, 2003; Oh et al., 2018) and note that other landmarking methods can also be used to learn facial expression.

### 3.3. Deep Learning

**Figure 2:**
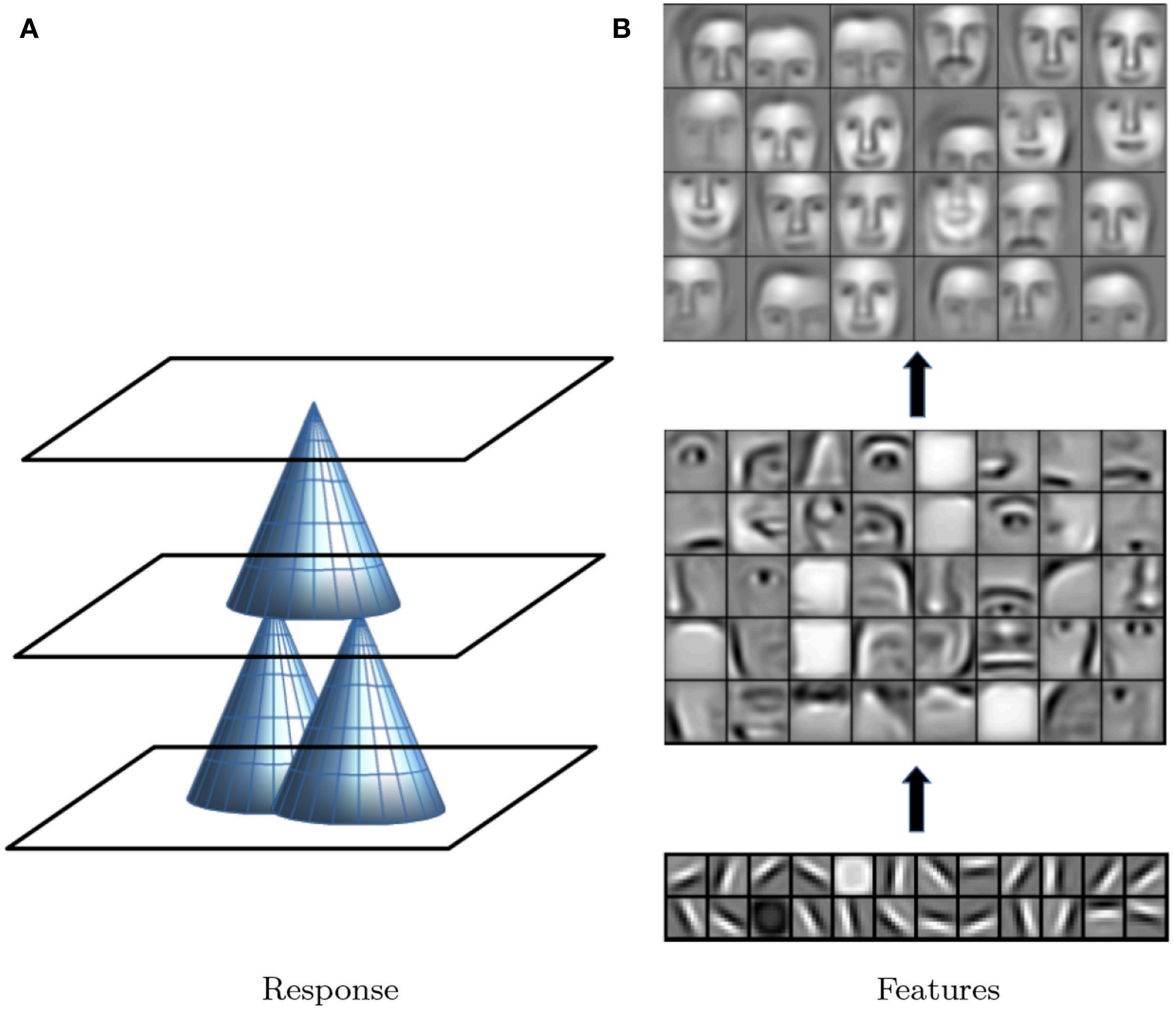
**(A)** For three example responses, each cone represents an image patch on a lower level on which the response of the level above depends. Through the middle layer, the upper layer depends on a wider input field in the lower level than the middle level. **(B)** Cartoons of input image patches that would create maximal responses in layers of CNNs (first layer at the bottom, last at the top). The first layer responds to edges, plain areas or point elevations in all cases. The second layer is specific to the learning objective, e.g., facial anatomical features such as eyes, noses, or mouths. The final layer recognizes objects as a whole. With permission from Lee et al. (2009).

Deep learning and deep neural networks (DNN) are terms for learning algorithms involving many stacked layers of functions (or regressions) for which parameters are estimated to optimize a final learning objective (LeCun et al., 2015). For example, similar to the application of standard regression models, this can be used to classify a pixel in an image to be either a given landmark or not, or to predict a certain landmark coordinate. Statistically, deep learning can be best compared to stacking (Breiman, 1996), where several base models are built to predict an outcome. Then, a second model is built to predict the outcome again based on output from the base models thereby generating a two-layer prediction. Deep learning similarly uses the output of regressions (or more generally parametrized functions) on a lower level as input for higher levels to predict a final output. Usually more than two layers are used in DNNs. So-called convolutional networks (CNNs) are a variant of DNNs that is applied on image or voxel data. They make use of functions that only look at smaller image patches within an input image instead of the full image. In a sliding-window approach the same function is applied to every possible patch placement and the result is summarized in a new picture (each new pixel is the value of the function for the patch around the pixel from the input). This is similar to a template search where at each pixel the function would indicate how well the template matches at the current position. Higher layers repeat this procedure. As each layer looks at a patch from the previous input layer, higher level outputs depend on increasingly larger patches of the initial input image (Figure 2A). Intuitively, this is an important aspect, as features can be combined into increasingly larger units, say edges (first layer) are combined into structures like mouth and nose (second layer) and these are composed into faces (third layer). This concept is illustrated in Figure 2B. Every layer represents features needed to detect faces/landmarks at different levels of complexity. Which functions are needed to achieve these representations is learned during model training. This allows to use raw pixel data as input without pre-processing. For landmarking purposes, CNNs are trained on manually labeled input images so that the resulting model can predict landmark coordinates for new input data.

Disadvantages of CNNS are that the flexibility of the models also requires large training samples, the design of the network architecture is largely empirical, and the fitting of the models requires careful tuning (Goodfellow et al., 2016).

Deep networks for landmarking have been well established. A CNN using raw pixel information of 2D images was augmented by components estimating pose, sex, and other aspects using a so-called multi-tasking approach (Zhang et al., 2016). The input was reduced to 40 × 40 gray scale images and four layers were used. Still the method had to rely on 10,000 training images. A similar approach using residual learning was proposed for 2D data (Ranjan et al., 2017). Residual learning is a strategy to mitigate the cost in terms of training sample size with respect to the depth of the network. Instead of using only outputs of a lower layer, residual networks copy the input of the previous layer as well (He et al., 2016). This is analogous to statistical models when main effects, i.e., the raw data should always be included in the model. This strategy was used to combine 2D and 3D data (Bulat and Tzimiropoulos, 2017) in an effort to further alleviate the limitation in the availability of 3D surface data. Augmenting training data by synthetic training data is another typical strategy to increase available training data.

When only scarce training data is available and data combination strategies are not an option, so-called transfer learning is yet another way to train deep networks. In this case, a pre-trained network is used and the last or last few layers are removed (Burlina et al., 2017). This latter application involves image classification using GoogleNet (Szegedy et al., 2015) as the source network. A new classifier can then be trained on few training examples by re-adding a final classification layer. The intuition behind this approach is that the source network has learned relevant features that can also be used for the classification problem at hand. A non-standard approach combines convolutional networks with a PDM (Zadeh et al., 2017). Landmarking of volumetric data has also been addressed (Zheng et al., 2015).

### 3.4. Other Models

In this section, we give a brief overview of some alternative approaches. An interesting approach is represented by *generative models*. Conceptually, a model is used to render the image of a face. This model is controlled by few parameters. Each set of parameters can be rendered into a face for which the landmark coordinates are known. These models therefore have a one-by-one correspondence between parameters and landmark coordinates. By generating an image that best resembles a given face in 2D or 3D, all landmarks are directly identified by reading them off of the model (Blanz and Vetter, 1999, 2003). Such models have not received much attention recently. Deep networks can also be used generatively to produce facial renderings but do not produce landmarks in a standard setting (Goodfellow et al., 2016).

*Agent based models* consider landmarking as a task of finding the correct path to a landmark from a starting position where reinforcement learning can be used in combination with deep networks (Ghesu et al., 2018; Alansary et al., 2019). The search strategy as opposed to the landmarking accuracy itself is optimized and landmarking is a by-product.

Many *heuristic* methods have been proposed focusing on specific properties of the data set at hand (for example He et al., 2012; Wilamowska et al., 2012; Guo et al., 2013; Peng et al., 2013). A heuristic step for detecting anatomical landmarks can be combined with dense surface registration (Guo et al., 2013; Peng et al., 2013). Such methods are characterized by the fact that the introduction of new landmarks would require changes to feature representations or the underlying model.

Atlas-based methods define a single or a few instances of faces (or geometric objects in general) which are annotated with additional information such as landmarks. If the atlas is transformed to match the target instance, the annotations transfer as well and establish landmarks in the target image (Li et al., 2017).

## 4. Symmetry Registration

Symmetry (or asymmetry) estimation addresses an important aspect of facial data. The degree of symmetry is an important property of a face having a connection with attractiveness (Scheib et al., 1999; Leyvand et al., 2008) and an impression of dysmorphia which in turn is linked with genetic syndromes (Winter, 1996; Thornhill and Møller, 1997).

The process of symmetry registration is to establish left-right correspondence between either anatomic landmarks, pseudolandmarks, or pixels.

A first approach uses localized, weighted correlation of image patches in 2D to find corresponding pixels (Kalina, 2012). In this approach, every image patch on one side forms a template for the other side. This approach can be applied to 3D but needs face registration to avoid distortions when 3D surface models are projected to 2D.

A second approach is to see symmetry registration as a landmarking problem. Instead of establishing correspondence across scans, correspondence between halves of the face is sought. To this end, one approach is to first mirror the scan with respect to the x-coordinate (first axis) after registering the face and then to find landmarks being close to each other when comparing the original and mirrored scan (Claes et al., 2011; Taylor et al., 2014).

Having registered symmetry of a face results in a multivariate data sets with up to 10^5^ dimensions. Perception of asymmetry by humans, however, is likely to rely on only a few dimensions. Some results are available to connect raw asymmetry as calculated from registration with perceived asymmetry. Facial symmetry perception may vary from observer to observer (Scheib et al., 1999). Also different parts of the face contribute differently to the perception of symmetry (Hwang et al., 2012; Storms et al., 2017).

## 5. Morphometric Analysis and Reliability

### 5.1. Procrustes Alignment

After landmarks have been placed in a given image, landmark coordinates have to be standardized across the sample. Each set of landmarks is represented by a graph. The standard approach is to use a generalized Procrustes analysis which chooses the so-called Procrustes mean shape as a graph (Kendall, 1989) so that distance to the graphs in the sample are minimized after they have been translated, scaled, and rotated in an optimal way (Gower, 1975) (generalized Procrustes analysis; GPA). The distance is measured for corresponding landmarks. Procrustes residuals—the difference of the standardized graph of a sample and the Procrustes mean shape—have the advantage of being independent (Dryden and Mardia, 1998). This is in contrast with other methods of standardization such as Bookstein coordinates, where a pair of landmarks is used to define translation, scaling, and rotation to bring this pair of coordinates into a standard position (Dryden and Mardia, 1998).

### 5.2. Transformations

Coordinates of landmarks and pseudo-landmarks offer a raw quantification of the face. Often coordinates are transformed to enter statistical analyses. Most studies investigate pair-wise distances, fewer look at angles and areas of triangles as derived from a triangulation. The variance of the transformed values is usually larger than the variance of the coordinates. If, for example, two coordinates are subtracted *D* = *X*_2_ − *X*_1_, the variance of *D* is *Var*(*D*) = *Var*(*X*_1_) + *Var*(*X*_2_) + 2*Cov*(*X*_1_, *X*_2_). For independent coordinates, the variances add up, positively correlated coordinates are resulting in a bigger variance. Applying transformations is therefore a tradeoff between generating useful features and introducing more variance. The variance can be decomposed in a part due to true, biological variation, and in measurement error. If the transformation is meaningful, the signal is not necessarily attenuated and the resulting features might better correlate with the outcome. In syndrome classification problems transformations seem to provide useful information (Balliu et al., 2014; Kraemer et al., 2018). Many GWASs and candidate gene studies use these transformations as outcomes (Boehringer et al., 2011b; Liu et al., 2012; Paternoster et al., 2012; Adhikari et al., 2016; Cole et al., 2016; Shaffer et al., 2016; Lee et al., 2017). In these settings, the transformations are fixed, i.e., independent of the data. The case when transformations are estimated is discussed in section 7.

### 5.3. Dense Surface Models

3D surface data as produced by commercial scanners (Boehnen and Flynn, 2005), 2016 is exported as a triangulated 3D-graph plus an accompanying texture that can be used to re-render the surface. For data analysis it is desirable to interpret the vertices of the graph as (dense) landmarks and establish correspondence between scans. This can dramatically augment the data set in that 10^4^–10^5^ landmarks are produced.

One approach is based on first aligning faces, followed by a step that identifies close vertices by a nearest neighbor approach (Hutton et al., 2001). The alignment first uses a GPA to align faces using a linear transformation based on anatomical landmarks. In a second step, anatomical landmarks are aligned exactly using a warping technique (Bookstein, 1996). Intuitively, the one graph of anatomical landmarks has to be bent into the shape of the second graph. This movement can be quantified, for example by a bending energy (Bookstein, 1996), and the movement minimizing the criteria is chosen (thin-plate splines, TPS). This movement on the anatomical landmarks drags along the pseudo-landmarks in between. After this alignment, the correspondence can be established as described above using the nearest neighbors. As always when two faces are aligned, a common reference has to be chosen to align all samples, a common choice being one of the samples. The number of aligned dense landmarks varies depending on how many vertices are available from the initial scans which can be a disadvantage. Sub-sampling vertices from samples is one strategy to make the number of dense landmarks comparable across samples.

### 5.4. Pseudo-Landmarks

Pseudo-landmarks are mathematically defined landmarks, for example a landmark halfway between two anatomical landmarks (Dryden and Mardia, 1998). This can be used to control the number of landmarks in a dense model. When the surface of the face is described with mathematical functions, an arbitrary number of landmarks can be derived from such a representation (Gilani et al., 2015). Similar approaches are based on mathematical functions that interpolate the surface between anatomical landmarks. For example, the functions may be chosen to make the curvature of these functions similar across samples (or a reference template). In a second step, one can sample landmarks from the resulting functional representation (Litke et al., 2005; Grewe and Zachow, 2016).

So called variational implicit functions have been used in pseudo-landmark alignment (Claes et al., 2005). In this approach, again a continuous function is found that interpolates the graph representing the scan for all in-between points. The approach allows to control the smoothness of the interpolation. To align faces, one starts by transforming anatomical landmarks between the faces. The functions that interpolate the in-between points have to be analogously transformed. Once this transformation is found, correspondence between all points is established (Turk and O'brien, 1999a,b).

## 6. Reliability and Heritability

Reliability denotes the agreement of several measurements. Reliability can be further distinguished into repeatability, the agreement of repeated measurements with the same method, and reproducibility, the agreement of different methods (Petrie and Sabin, 2013) when measurements are taken under similar conditions. Replication efforts in genetic studies of facial traits therefore require reproducibility of quantification. Reliability of manual landmarking efforts have been evaluated in some studies (Fagertun et al., 2014; Katina et al., 2015; de Jong et al., 2018a). The agreement between raters was consistently reported to be between 1 and 2 mm across landmarks. The most detailed of the above studies (Katina et al., 2015) performed multiple repeats (within/across days/raters) and could show that repeatability (i.e., the consistency of a single rater) is lower than 1 mm for 18 out of 21 landmarks but also that the combined error across raters, time points and image presentations falls into the range mentioned above. Systematic evaluation with many—i.e., more than 30—human raters is missing. Also, the absolute calibration of landmarking positions has mostly been insufficiently described, which makes generalizations difficult.

Heritability estimation has been an early interest in facial research (Hunter et al., 1970; Nakata et al., 1976; Hoskens et al., 2018; Richmond et al., 2018). Heritability denotes the proportion of variation in an outcome that is attributable to genetic variation. Heritabilities can be calculated for additive genotype contribution (narrow sense) and a general genotypic model (broad sense). Narrow sense heritability can be estimated in families when only phenotype data is available. Heritability can be used to evaluate the reliability of a landmarking method by comparing heritability as estimated from landmarks attained with one method to that of a benchmark method which might be another landmarking method or a previous version of the same method. When measurement error is reduced, total variation should be reduced and the proportion explained by genetic contributions should increase. This approach has been used to evaluate landmarking methods (de Jong et al., 2018a,b).

**Figure 3:**
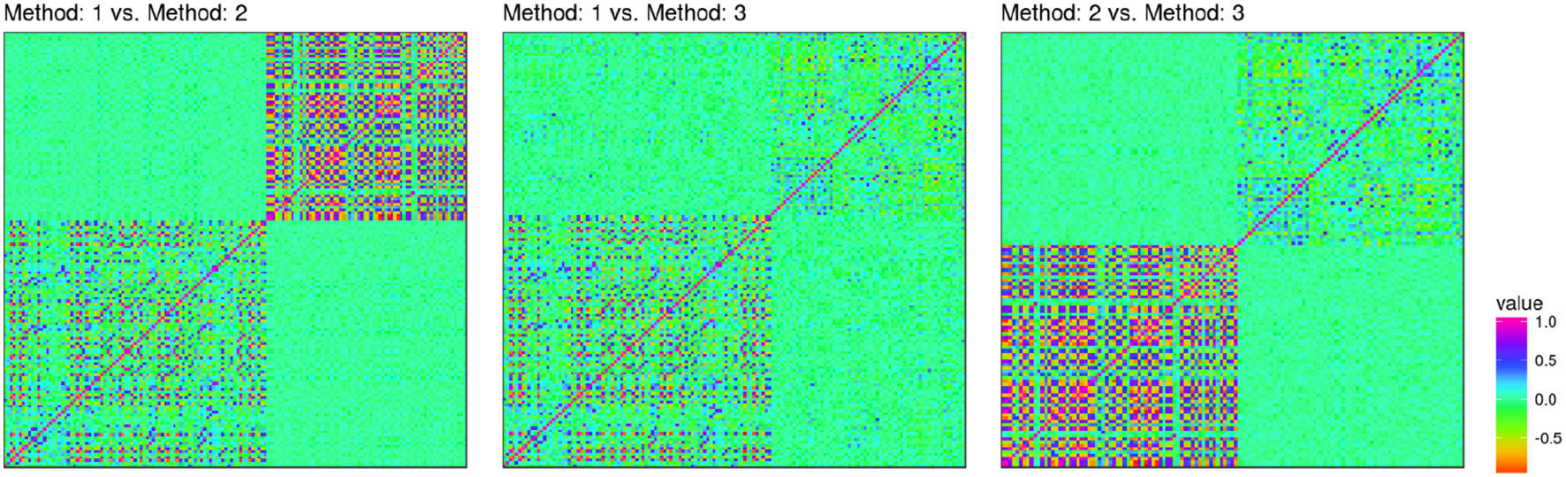
Correlation heatmap comparing 3d-coordinates of three landmarking methods on an identical data set (Twins UK). The lower left block corresponds to (*x, y, z*)-coordintes of the first methods, the upper right block to those of the second method. Off-diagonal blocks correspond to correlations between methods. Method 1 (29 landmarks) and method 3 (21 landmarks) are automatic methods. Method 2 is manual (22 landmarks).

Reproducibility determines the chance of study replication. It can be measured by the correlation between measurements of different methods. Although measurement agreement, i.e., reliability, is often evaluated using Bland-Altman plots (Bland and Altman, 1986), correlation has an important statistical implication. Statistically, *R*^2^, i.e., the squared correlation can be seen as the (percentage) loss in sample size when one measurement method is used instead of another one. An *R*^2^ can be defined for general likelihood models, for binary, linear, survival and count outcomes, among others (Orchard and Woodbury, 1972; Louis, 1982) which is known as the missing information principle. The “other” measurement can be seen as missing data that has to be inferred from the measurement at hand. This relationship is also known from the design of SNP panels as the omission of a particular SNP can be seen as missing data (Pritchard and Przeworski, 2001). For example, the consequences of a correlation of ~ 0.7 means that effective sample size is reduced to a half, when a particular measurement is replicated in an independent cohort. Figure 3, shows correlations of coordinates of an informal comparison as conducted on a data set as used in a previous study involving the Visigen consortium (de Jong et al., 2016), comparing two automatic and one manual landmarking approach on an identical set of individuals. In this comparison, landmark positions were standardized using Procrustes analysis within each study. On this data, pairwise correlations between all landmark coordinates (*x, y, z*) were computed and used as a measure to judge inter-method variability, i.e., reproducibility. The maximal correlation was around ~ 0.6. While informal, these results indicate high variability between landmarking efforts. Regarding heritability, the best automatic methods performed similar to manual landmarking (*h*^2^ ≈ 0.6).

### 6.1. Implications of Landmark Definition

As more cohorts are phenotyped for facial traits and data sets are aggregated either through meta- or joint analysis, it is worthwhile to consider reproducibility a bit further. The measurement error of one method as compared to another has two components: bias and variance. The bias can be seen as the differences in landmark definitions. While anatomical landmarks are defined descriptively, the actual definition used in a study depends on human raters. A large majority of studies relies on human raters either directly through complete manual labeling (Boehringer et al., 2011b; Paternoster et al., 2012) or indirectly in the sense that an automated method is trained on human labeled training images (Liu et al., 2012). The bias due to implicitly varying landmark definitions can be evaluated by comparing sets of landmarks defined in a different way. It has been suggested that definitions based on curvatures(Bowman et al., 2015; Katina et al., 2015) might have less variability and might therefore induce less inter-study bias. Curvatures have also been used in heritability evaluation (Tsagkrasoulis et al., 2017) and seem promising. We return to the problem of landmark definitions in the discussion.

## 7. Global Quantification

Using local or morphometric descriptors in genetic analyses has the disadvantage of leading to a combinatorial expansion of features. An attractive alternative is to employ dimension reduction to make the analysis more straightforward and potentially more meaningful. We discuss classic linear, non-linear, and generative approaches in addition to principal components of heritability.

### 7.1. Variance Based Approaches

Principal component analysis (PCA) is one of the most widely used dimension reduction technique (Jolliffe, 2005) and has been employed in many facial studies (Hutton et al., 2001; Hammond et al., 2005, 2012; Boehringer et al., 2006; Vollmar et al., 2008). The first principal component (PC) is defined as a linear combination of raw data *x*_*ij*_, with individual *i* and feature *j* (for example a coordinate). The PC score of individual *i* is $z_{i}=\overset{p}{\underset{j=1}{\sum }}w_{j}x_{ij}$. The weights, called loadings, *w*_*j*_ form a unit length vector and are chosen to maximize the variance of the scores *z*_*i*_. Higher order PCs are again unit length linear weight vectors maximizing variance subject to the constraint that they are orthogonal to all lower order PCs. One important difference to the transformations discussed in section 5 is that the transformation is also random as the loadings are estimated from the data. Both empirical (Molinaro et al., 2005; Boehringer et al., 2011a) and theoretical (Bai and Silverstein, 2010) considerations imply that PCA has the largest influence on reproducibility by contributing variability into the analysis that is larger than that induced by measurement error. This relationship can be evaluated using cross-validation (CV) (Hastie et al., 2001), i.e., a form of data splitting where one part of the data is used for model fitting and the left out part is used for evaluation (test data). This data-splitting mimics a replication experiment. In a study where syndromes were predicted from 2D facial data (Boehringer et al., 2011a), the difference in prediction accuracy when first PCA was performed on the whole data before data splitting and second when PCA was performed on the training data and loadings were carried over to the test data was 60% compared to 21%.

There are more generic multivariate techniques that have been employed in facial analysis. Among them are partial least squares (PLS) (Garthwaite, 1994), canonical correlation (Hotelling, 1936), and discriminant analysis (Fisher, 1936). To address the problem of high variability due to loadings estimation, so-called sparse methods have been introduced. Instead of using all input features, the scores are only computed on a subset of input features by setting many loadings (weights *w*_*i*_) to zero. Technically, penalization is used (Tibshirani, 1996) and sparse PCA (Zou et al., 2006), sparse PLS (Witten et al., 2009), sparse canonical correlation (Witten et al., 2009) and other sparse methods have received attention. Their effect on reproducibility has not yet been systematically evaluated on facial data.

Historically, one of the early facial analysis applied PCA on the pixel data of an image, interpreted as a single long vector of gray values (Turk and Pentland, 1991). This approach has been used for face recognition but has not been employed in genetic association studies as the variability inherent in the estimation of a PCA make replication difficult.

### 7.2. Manifold Learning

The methods mentioned in the previous section are all based on linear combinations of input and/or outcome data. It is possible to employ non-linear methods. When an outcome is to be predicted from facial input features, non-linear regression techniques can be used. These include generalized additive—spline based—models (Hastie and Tibshirani, 1990) and support vector regression (SVR). SVR has been used to predict facial attractiveness, and subsequently “beautify” a given face (Leyvand et al., 2008).

**Figure 4:**
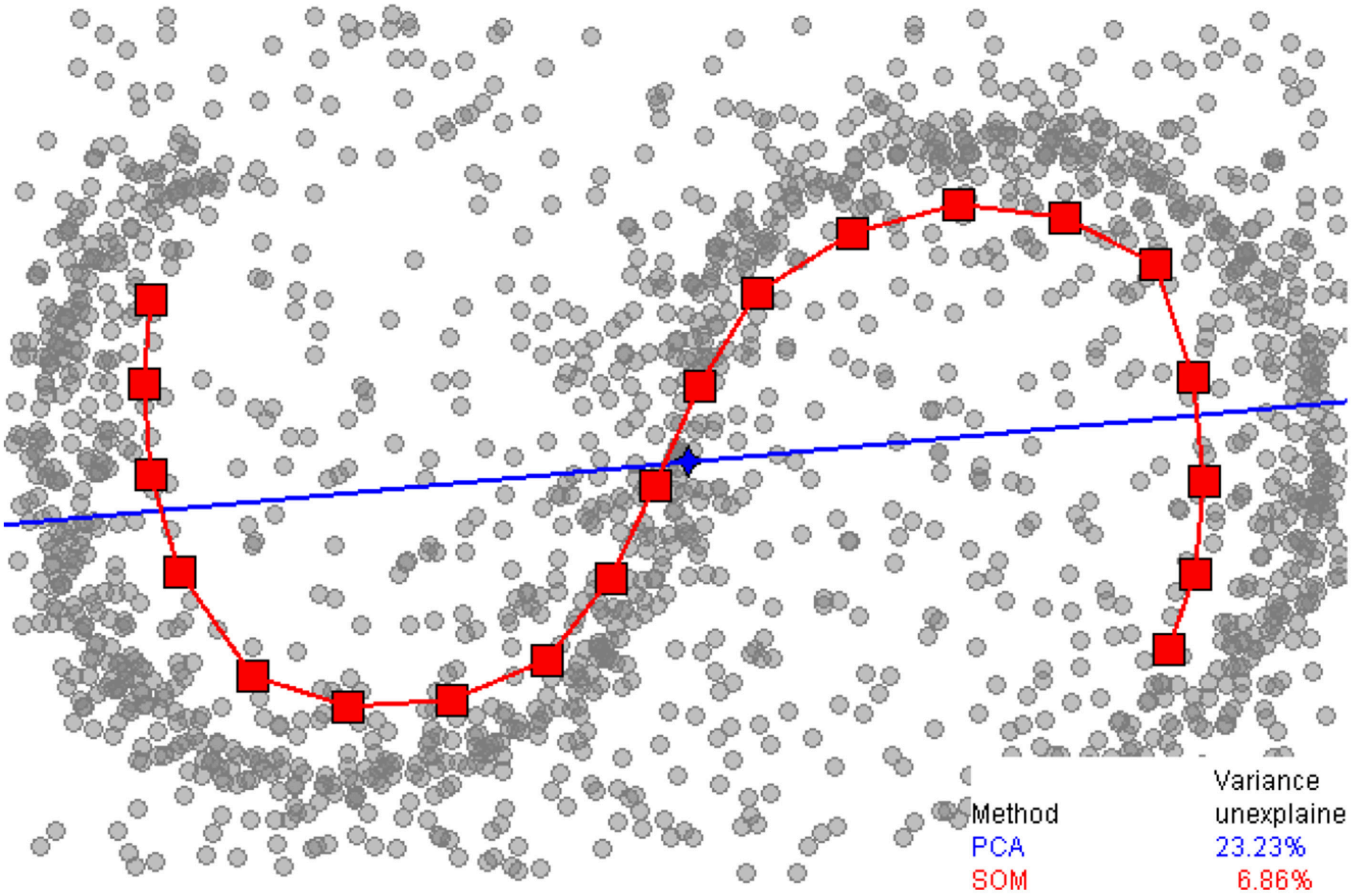
Example of global (PCA) and local (self-organizing map; SOM) dimension reduction (2018). PCA approximates the data along the blue line, whereas SOM estimates the red curve. Source: wikipedia.

It is also possible to perform dimension reduction on facial data alone using non-linear transformations. In three dimensions, the reduction would not lead to a flat plane or straight line but rather to a curved 2D-surface or 1D-curve, winding through three-dimensional space. The estimation of these transformations is known as generalized PCA or manifold learning (Vidal et al., 2016). Recently, local embedding techniques have received attention. The motivation for these techniques is given by the sinusoid data in Figure 4. The proper description of a data point would be a single number: the length along the curve that a given point is closest to. PCA on the other hand would not “understand” the data. The data has the biggest extent along the x-direction, leaving the data almost unchanged after PCA, except for a small rotation. If only a single PC were chosen much of the information in the data would be discarded. t-SNE (van der Maaten and Hinton, 2008) is one such method and has been used for facial analysis (Booth et al., 2016). A disadvantage of t-SNE is that is does not allow to embed new images into the same coordinate system which is due to the algorithm used. t-SNE can therefore only be used for visualization. Local linear embedding (Roweis and Saul, 2000) and local multidimensional scaling (Tenenbaum et al., 2000) are alternatives without this limitation. The latter study contains an application to synthetic facial data.

The DNN approach to manifold learning is called autoencoding (Goodfellow et al., 2016). In this case, the network is used in two directions. When values are available at the output layer, they are run down the network again to produce potential input data, compatible with values at the output layer. The actual input can be compared with the potential input and the network will be trained to minimize these discrepancies. Deep autoencoders have been employed in the landmarking problem but it is unclear whether they can outperform more tailor-made models (Zhang et al., 2014).

### 7.3. Principal Components of Heritability

An attractive way to perform dimension reduction in a genetic application is to take into account heritability. This approach requires family data and is based on the fact that variation in any outcome can be decomposed into intra-family and inter-family variation which add up to the total variation of the variable, say a distance. By rescaling the variable such that the intra-family variation is 1, the total variation becomes a measure for heritability, *i.e*. inter-family variation. In a multivariate setting, this rescaling becomes a matrix multiplication (Ott and Rabinowitz, 1999). Finding the largest variation in the rescaled data becomes an application of PCA. The resulting component is called principal component of heritability (PCH). The initial approach can only deal with families of identical structure, for example sibships. Another problem is the variability of the required estimations, the rescaling and the PCA which become unstable for high-dimensional data. The dimensionality problem can be addressed by penalization (Wang et al., 2007; Oualkacha et al., 2012). The method has also been generalized to heterogenous families (Oualkacha et al., 2012). PCHs only represent narrow sense heritability, i.e., explained variance due to additive genetic effects. PCHs have not been systematically applied to facial data.

## 8. Discussion

Automatic quantification of facial traits is in a state of rapid development. Many state-of-the-art methods are competitive with human raters for many anatomical landmarks. Still methods differ in important properties which we summarize in Table 1. Template based methods work with very small training efforts (de Jong et al., 2018b) but require more intense pre-processing than ASMs (Milborrow and Nicolls, 2014) or DNNs (Bulat and Tzimiropoulos, 2017). Pre-trained ASMs and DNNs are available but a recent comparison showed that re-training a template based method for a data set at hand outperforms the pre-trained model (de Jong et al., 2016). As pre-trained models are based on larger and larger samples, this situation might change and it will be important to keep evaluating the gain achieved by re-training. One advantage of using pre-trained models would be the availability of more homogeneous data across studies, which would help replication efforts.

**Table 1:** Comparison of landmarking methods.

**Method class**	**Training complexity**	**Robustness**	**Specific advantages**	**Example implementation**
Template based	Low	Low without pre-processing	Quick to deploy	https://opencv.org
Active shape	Moderate to high	Good to high	Facial expression analysis	http://www.milbo.users.sonic.net/stasm
Deep learning	High	High	No pre-processing required	https://www.adrianbulat.com/face-alignment
Generative	Moderate	–	Rendering of potential faces	–

*Low, moderage, high complexity correspond to <100, 100-1,000, >1,000 training samples, respectively. Robustness refers to performance under varying image conditions such as lighting and pose and assumes such variation is represented in the training sample*.

Face recognition plays an important role in social media, biometric access control, and increasingly in law enforcement (Lynch, 2018) and is therefore also a field in rapid development. It needs to detect the general shape of a face on an image and needs to learn unique features identifying an individual independently of confounding factors such as lighting, pose, and camera properties. This application has been pursued by commercial entities such as Google and is subject to intense research. The most promising implementations make use of deep neural networks (DNN) and learn face representations implicitly through parameters of the network (Schroff et al., 2015). These models predate DNN models used for landmarking. Landmarking models share large portions of their architecture with pure face recognition models and developments in either class of models will influence the other.

All models discussed that provide anatomical landmarks rely on human raters at some point. This implies that there is an implicit difference in landmark definitions between studies, as human raters do only agree on landmark definitions to some degree (section 6.1). An open question is the automatic definition of landmarks. One possible approach is to use manifold learning that needs to describe the sample sparsely, for example through an information criterion (Cover and Thomas, 2012). As experiments on alternative landmark definitions show (Katina et al., 2015), it seems promising to look for better definitions to improve replication efforts.

As pointed out elsewhere (Evans, 2018) it is desirable to quantify a face globally. Individual morphometric features, say distances, are unlikely to be important in face perception. More global patterns determine the importance of facial appearance and are therefore arguably also likely to be under common genetic control. An interesting strategy is to analyze features at the global and local level simultaneously. Using hierarchical spectral clustering, a recent study could demonstrate effects of genetic loci on different levels (Claes et al., 2018). The idea is to start with an aggregation of the full facial landmark data and analyze genetic association with respect to this summary. In following steps, landmark data is partitioned into subsets and analyzed in an analogous manner within the subsets. This subsetting can be repeated to demonstrate genetic effects on specific facial sub-regions and thereby gives a more comprehensive view of genetic associations as compared to other GWAS analyses.

Intuitively, facial appearance follows a local pattern: there is a smooth transition between all possible pairs of faces and local embedding techniques should therefore be well suited to cover biological variation. With very large sample sizes, the randomness of the transformation into the manifold can be reduced and models based on large external data sets could be used. Non-genetic facial databases reach more than 100,000 individuals (Bulat and Tzimiropoulos, 2017) and are a potential source for such models. However, apart from biological variation there is also technical variation that might not follow a local pattern (Gagnon-Bartsch and Speed, 2012). It is therefore likely that manifold learning has to take into account both local and global structure (McInnes and Healy, 2018). Both empirical and theoretical work is needed to solve this issue

For the technological measurement process, rapid developments are expected. Lensless imaging or multiple cameras on a mobile phone are likely to create new data sources for facial 3D scans (Ozcan and McLeod, 2016) which will require adaptations in quantification. Combination with volumetric data such as CT or MRI data might help to improve quantification.

We would also like to point out the importance of applications in the development of quantification methodology. Before GWASs were performed, applications in syndrome classification were introduced. First, these covered more common syndromes as single classes, such as Microdeletion 22q11.2, Fragile X, Noonan, Smith-Lemli-Opitz, Cornelia de Lange, and others (Loos et al., 2003; Hammond et al., 2005; Boehringer et al., 2006). The focus of this field has now shifted to identifying mutations within syndrome classes (Gurovich et al., 2019). GWASs could make use of landmarking techniques used for syndrome classification but needed adaptations. More automation was necessary and as landmarking accuracy directly affects statistical power, accuracy became a new focus. This was not necessarily the case in syndrome classification as facial appearance might be different enough across groups to tolerate noise. A similar situation exists with respect to asymmetry, which has been shown to be important in conditions like fetal alcohol syndrome and autism (Hammond et al., 2008; Klingenberg, 2008; Klingenberg et al., 2010). A first GWAS on facial asymmetry has been published and shows some overlap with disease associated genes (Rolfe et al., 2018).

In conclusion, accuracy of facial quantification is of critical importance for both power and reproducibility of genetic studies with respect to facial traits. Power loss can be dramatic in replication efforts even when heritabilities estimated from quantification methods are similar on average (section 6). Standardizing the analysis could help and would open the door for easy re-analyses once improvements in quantification have been found. It is biologically plausible that facial appearance has a similar or larger genetic complexity as compared to body height (Allen et al., 2010). With appropriate quantification, it should be possible to find the corresponding genes.

## Author Contributions

SB and MdJ conception of the manuscript, writing, and approval of the manuscript.

### Conflict of Interest Statement

The authors declare that the research was conducted in the absence of any commercial or financial relationships that could be construed as a potential conflict of interest.

## References

[B1] (2016). 3dMD–3D imagingsystems and software.

[B2] (2018). Nonlinear Dimensionality Reduction. Page Version ID:848551806.

[B3] Adhikari K. Fuentes-Guajardo M. Quinto-Sánchez M. Mendoza-Revilla J. Chacón-Duque J. C. Acuña-Alonzo V.. (2016). A genome-wide association scan implicates *DCHS2, RUNX2, GLI3, PAX1* and *EDAR* in human facial variation. Nat. Commun. 7:11616. 10.1038/ncomms11616 27193062 PMC4874031

[B4] Alansary A. Ozan O Yuanwei L. Loic L. F. Benjamin H. Ghislain V.. (2019). Evaluating reinforcement learning agents for anatomical landmark detection. Medical Image Analysis 53, 156–164. 10.1016/j.media.2019.02.007 30784956 PMC7610752

[B5] Allen H. L. Estrada K. Lettre G. Berndt S. I. Weedon M. N. Rivadeneira F. (2010). Hundreds of variants clustered in genomic loci and biological pathways affect human height. Nature 467, 832–838. 10.1038/nature09410 20881960 PMC2955183

[B6] Bai Z. Silverstein J. W. (2010). Spectral Analysis of Large Dimensional Random Matrices, Vol 20. New York, NY: Springer.

[B7] Balliu B. Würtz R. P. Horsthemke B. Wieczorek D. Böhringer S. (2014). Classification and visualization based on derived image features: application to genetic syndromes. PLoS ONE 9:e109033. 10.1371/journal.pone.0109033 25405460 PMC4236018

[B8] Bland J. M. Altman D. G. (1986). Statistical methods for assessing agreement between two methods of clinical measurement. Lancet 1, 307–310. 10.1016/S0140-6736(86)90837-8 2868172

[B9] Blanz V. Vetter T. (1999). “A morphable model for the synthesis of 3D faces,” in Proceedings of the 26th Annual Conference on Computer Graphics and Interactive Techniques (Los Angeles, CA: ACM Press; Addison-Wesley Publishing Co.), 187–194.

[B10] Blanz V. Vetter T. (2003). Face recognition based on fitting a 3D morphable model. IEEE Trans. Pattern Anal. Mach. Intell. 25, 1063–1074. 10.1109/TPAMI.2003.1227983

[B11] Boehnen C. Flynn P. (2005). “Accuracy of 3D scanning technologies in a face scanning scenario,” in Fifth International Conference on 3-D Digital Imaging and Modeling (3DIM'05) (Ottawa, ON), 310–317.

[B12] Boehringer S. Guenther M. Sinigerova S. Wurtz R. P. Horsthemke B. Wieczorek D. (2011a). Automated syndrome detection in a set of clinical facial photographs. Am. J. Med. Genet. Part A 155, 2161–2169. 10.1002/ajmg.a.34157 21815261

[B13] Boehringer S. van der Lijn F. Liu F. Günther M. Sinigerova S. Nowak S.. (2011b). Genetic determination of human facial morphology: links between cleft-lips and normal variation. Eur. J. Hum. Genet. 19, 1192–1197. 10.1038/ejhg.2011.110 21694738 PMC3198142

[B14] Boehringer S. Vollmar T. Tasse C. Wurtz R. P. Gillessen-Kaesbach G. Horsthemke B.. (2006). Syndrome identification based on 2d analysis software. Eur. J. Hum. Genet. 14, 1082–1089. 10.1038/sj.ejhg.5201673 16773127

[B15] Bookstein F. L. (1996). “Shape and the information in medical images: a decade of the morphometric synthesis,” in Proceedings of the Workshop on Mathematical Methods in Biomedical Image Analysis (San Francisco, CA), 2–12.

[B16] Booth J. Roussos A. Zafeiriou S. Ponniah A. Dunaway D. (2016). “A 3D morphable model learnt from 10,000 faces,” in Proceedings of the IEEE Conference on Computer Vision and Pattern Recognition (Las Vegas, NV), 5543–5552.

[B17] Bowman A. W. Katina S. Smith J. Brown D. (2015). Anatomical curve identification. Comput. Stat. Data Anal. 86, 52–64. 10.1016/j.csda.2014.12.007 26041943 PMC4394146

[B18] Bradski G. (2000). The OpenCV Library. Dr. Dobb's Journal of Software Tools. Available online at: https://github.com/opencv/opencv/wiki/CiteOpenCV

[B19] Breiman L. (1996). Stacked regressions. Mach. Learn. 24, 49–64.

[B20] Bulat A. Tzimiropoulos G. (2017). “How far are we from solving the 2D & 3D Face Alignment problem? (and a dataset of 230,000 3D facial landmarks),” in International Conference on Computer Vision (Venice).

[B21] Burlina P. Pacheco K. D. Joshi N. Freund D. E. Bressler N. M. (2017). Comparing humans and deep learning performance for grading amd: a study in using universal deep features and transfer learning for automated amd analysis. Comput. Biol. Med. 82, 80–86. 10.1016/j.compbiomed.2017.01.018 28167406 PMC5373654

[B22] Claes P. Roosenboom J. White J. D. Swigut T. Sero D. Li J.. (2018). Genome-wide mapping of global-to-local genetic effects on human facial shape. Nat. Genet. 50, 414–423. 10.1038/s41588-018-0057-4 29459680 PMC5937280

[B23] Claes P. Vandermeulen D. Van Gool L. Suetens P. (2005). “Partial surface integration based on variational implicit functions and surfaces for 3d model building,” in Fifth International Conference on 3-D Digital Imaging and Modeling, 3DIM 2005 (Ottawa, ON), 31–38.

[B24] Claes P. Walters M. Vandermeulen D. Clement J. G. (2011). Spatially-dense 3d facial asymmetry assessment in both typical and disordered growth. J. Anat. 219, 444–455. 10.1111/j.1469-7580.2011.01411.x 21740426 PMC3187867

[B25] Cole J. B. Manyama M. Kimwaga E. Mathayo J. Larson J. R. Liberton D. K.. (2016). Genomewide association study of African children identifies association of SCHIP1 and PDE8a with facial size and shape. PLoS Genet. 12:e1006174. 10.1371/journal.pgen.1006174 27560698 PMC4999243

[B26] Cootes T. F. Taylor C. J. Cooper D. H. Graham J. (1995). Active shape models-their training and application. Comput. Vis. Image Understand. 61, 38–59. 10.1006/cviu.1995.1004

[B27] Cover T. M. Thomas J. A. (2012). Elements of Information Theory. Hoboken, NJ: John Wiley & Sons.

[B28] de Jong M. A. Gül A. de Gijt J. P. Koudstaal M. J. Kayser M. Wolvius E. B.. (2018a). Automated human skull landmarking with 2d Gabor wavelets. Phys. Med. Biol. 63:105011. 10.1088/1361-6560/aabfa0 29676286

[B29] de Jong M. A. Hysi P. Spector T. Niessen W. Koudstaal M. J. Wolvius E. B.. (2018b). Ensemble landmarking of 3D facial surface scans. Sci. Rep. 8:12. 10.1038/s41598-017-18294-x 29311563 PMC5758814

[B30] de Jong M. A. Wollstein A. Ruff C. Dunaway D. Hysi P. Spector T.. (2016). An automatic 3d facial landmarking algorithm using 2d Gabor Wavelets. IEEE Trans. Image Process. 25, 580–588. 10.1109/TIP.2015.2496183 26540684

[B31] Dryden I. L. Mardia K. V. (1998). Statistical Shape Analysis. Chichester, NY: Wiley.

[B32] Edwards G. J. Taylor C. J. Cootes T. F. (1998). “Interpreting face images using active appearance models,” in Proceedings Third IEEE International Conference on Automatic Face and Gesture Recognition, 300–305.

[B33] Evans D. M. (2018). Elucidating the genetics of craniofacial shape. Nat. Genet. 50, 319–321. 10.1038/s41588-018-0065-4 29511283

[B34] Fagertun J. Harder S. Rosengren A. Moeller C. Werge T. Paulsen R. R.. (2014). 3d facial landmarks: inter-operator variability of manual annotation. BMC Med. Imaging 14:35. 10.1186/1471-2342-14-35 25306436 PMC4205300

[B35] Fasel B. Luettin J. (2003). Automatic facial expression analysis: a survey. Pattern Recogn. 36, 259–275. 10.1016/S0031-3203(02)00052-3

[B36] Fisher R. A. (1936). The use of multiple measurements in taxonomic problems. Ann. Eugen. 7, 179–188. 10.1111/j.1469-1809.1936.tb02137.x

[B37] Gagnon-Bartsch J. A. Speed T. P. (2012). Using control genes to correct for unwanted variation in microarray data. Biostatistics 13, 539–552. 10.1093/biostatistics/kxr034 22101192 PMC3577104

[B38] Garthwaite P. H. (1994). An interpretation of partial least squares. J. Am. Stat. Assoc. 89, 122–127. 10.1080/01621459.1994.10476452

[B39] Ghesu F. C. Georgescu B. Grbic S. Maier A. Hornegger J. Comaniciu D. (2018). Towards intelligent robust detection of anatomical structures in incomplete volumetric data. Med. Image Anal. 48, 203–213. 10.1016/j.media.2018.06.007 29966940

[B40] Gilani S. Z. Shafait F. Mian A. (2015). “Shape-based automatic detection of a large number of 3d facial landmarks,” in 2015 IEEE Conference on Computer Vision and Pattern Recognition (CVPR), 4639–4648.

[B41] Gomes J. Velho L. (2015). From Fourier Analysis to Wavelets, Vol 3. New York, NY: Springer.

[B42] Goodfellow I. Bengio Y. Courville A. (2016). Deep Learning. Cambridge, MA: MIT Press.

[B43] Gower J. C. (1975). Generalized procrustes analysis. Psychometrika 40, 33–51. 10.1007/BF02291478

[B44] Grewe C. M. Zachow S. (2016). “Fully automated and highly accurate dense correspondence for facial surfaces,” in European Conference on Computer Vision (Amsterdam: Springer), 552–568.

[B45] Guo J. Mei X. Tang K. (2013). Automatic landmark annotation and dense correspondence registration for 3d human facial images. BMC Bioinform. 14:232. 10.1186/1471-2105-14-232 23870191 PMC3724574

[B46] Gurovich Y. Hanani Y. Bar O. Nadav G. Fleischer N. Gelbman D.. (2019). Identifying facial phenotypes of genetic disorders using deep learning. Nat. Med. 25:60. 10.1038/s41591-018-0279-0 30617323

[B47] Hammond P. Forster-Gibson C. Chudley A. E. Allanson J. E. Hutton T. J. Farrell S. A.. (2008). Face–brain asymmetry in autism spectrum disorders. Mol. Psychiatry 13, 614–623. 10.1038/mp.2008.18 18317467

[B48] Hammond P. Hannes F. Suttie M. Devriendt K. Vermeesch J. R. Faravelli F.. (2012). Fine-grained facial phenotype –genotype analysis in Wolf–Hirschhorn syndrome. Eur. J. Hum. Genet. 20, 33–40. 10.1038/ejhg.2011.135 21792232 PMC3234504

[B49] Hammond P. Hutton T. J. Allanson J. E. Buxton B. Campbell L. E. Clayton-Smith J.. (2005). Discriminating power of localized three –dimensional facial morphology. Am. J. Hum. Genet. 77, 999–1010. 10.1086/498396 16380911 PMC1285182

[B50] Hastie T. Tibshirani R. Friedman J. (2001). The Elements of Statistical Learning: Data Mining, Inference, and Prediction. New York, NY: Springer.

[B51] Hastie T. J. Tibshirani R. J. (1990). Generalized Additive Models, Volume 43 of Monographs on Statistics and Applied Probability. London: Chapman & Hall.

[B52] He K. Zhang X. Ren S. Sun J. (2016). “Deep residual learning for image recognition,” in Proceedings of the IEEE Conference on Computer Vision and Pattern Recognition (Las Vegas, NV), 770–778.

[B53] He Q. Duan Y. Zhang D. (2012). “Automatic detailed localization of facial features,” in International Conference on Industrial, Engineering and Other Applications of Applied Intelligent Systems (Morioka: Springer), 1–9.

[B54] Hill A. Thornham A. Taylor C. J. (1993). “Model-based interpretation of 3D medical images,” in 4th British Machine Vision Conference, September 1993 (Guildford), 339–348.

[B55] Hoskens H. Li J. Indencleef K. Gors D. Larmuseau M. H. D. Richmond S.. (2018). Spatially dense 3d facial heritability and modules of co-heritability in a father-offspring design. Front. Genet. 9:554. 10.3389/fgene.2018.00554 30510565 PMC6252335

[B56] Hotelling H. (1936). Relations between two sets of variates. Biometrika 28, 321–377. 10.2307/2333955

[B57] Hunter W. S. Balbach D. R. Lamphiear D. E. (1970). The heritability of attained growth in the human face. Am. J. Orthodont. 58, 128–134. 10.1016/0002-9416(70)90066-7 5269761

[B58] Hutton T. J. Buxton B. F. Hammond P. (2001). “Dense surface point distribution models of the human face,” in Proceedings IEEE Workshop on Mathematical Methods in Biomedical Image Analysis (MMBIA 2001) (Kauai, HI).

[B59] Hwang H.-S. Yuan D. Jeong K.-H. Uhm G.-S. Cho J.-H. Yoon S.-J. (2012). Three-dimensional soft tissue analysis for the evaluation of facial asymmetry in normal occlusion individuals. Korean J. Orthod. 42, 56–63. 10.4041/kjod.2012.42.2.56 23112933 PMC3481973

[B60] Jolliffe I. (2005). Principal Component Analysis, 1st Edn. New York, NY: Wiley Online Library.

[B61] Kalina J. (2010). “Locating landmarks using templates,” in Nonparametrics and Robustness in Modern Statistical Inference and Time Series Analysis: A Festschrift in Honor of Professor Jana Jurecková (Beachwood, OH: Institute of Mathematical Statistics), 113–122.

[B62] Kalina J. (2012). Facial symmetry in robust anthropometrics. J. Forens. Sci. 57, 691–698. 10.1111/j.1556-4029.2011.02000.x 22150845

[B63] Kass M. Witkin A. Terzopoulos D. (1988). Snakes: active contour models. Int. J. Comput. Vis. 1, 321–331. 10.1007/BF00133570

[B64] Katina S. Mcneil K. Ayoub A. Guilfoyle B. Khambay B. Siebert J.. (2015). The definitions of three-dimensional landmarks on the human face: an interdisciplinary view. J. Anat. 228, 355–365. 10.1111/joa.12407 26659272 PMC4832301

[B65] Kendall D. G. (1989). A survey of the statistical theory of shape. Stat. Sci. 4, 87–99. 10.1214/ss/1177012582

[B66] Klingenberg C. P. (2008). Morphological integration and developmental modularity. Annu. Rev. Ecol. Evol. Syst. 39, 115–132. 10.1146/annurev.ecolsys.37.091305.110054

[B67] Klingenberg C. P. Wetherill L. Rogers J. Moore E. Ward R. Autti-Rämö I.. (2010). Prenatal alcohol exposure alters the patterns of facial asymmetry. Alcohol 44, 649–657. 10.1016/j.alcohol.2009.10.016 20060678 PMC2891212

[B68] Kraemer M. Huynh Q. B. Wieczorek D. Balliu B. Mikat B. Boehringer S. (2018). Distinctive facial features in idiopathic Moyamoya disease in Caucasians: a first systematic analysis. PeerJ 6:e4740. 10.7717/peerj.4740 29977664 PMC6029584

[B69] Lanitis A. Taylor C. J. Cootes T. F. (1997). Automatic interpretation and coding of face images using flexible models. IEEE Trans. Pattern Anal. Mach. Intell. 19, 743–756. 10.1109/34.598231

[B70] Lebow M. R. Sawin P. B. (1941). INHERITANCE OF HUMAN FACIAL FEATURES:a pedigree study involving length of face, prominent ears and chin cleft. J. Hered. 32, 127–132. 10.1093/oxfordjournals.jhered.a105016

[B71] LeCun Y. Bengio Y. Hinton G. (2015). Deep learning. Nature 521, 436–444. 10.1038/nature14539 26017442

[B72] Lee H. Grosse R. Ranganath R. Ng A. Y. (2009). “Convolutional deep belief networks for scalable unsupervised learning of hierarchical representations,” in Proceedings of the 26th Annual International Conference on Machine Learning (ACM), 609–616.

[B73] Lee M. K. Shaffer J. R. Leslie E. J. Orlova E. Carlson J. C. Feingold E.. (2017). Genome-wide association study of facial morphology reveals novel associations with FREM1 and PARK2. PLoS ONE 12:e0176566. 10.1371/journal.pone.0176566 28441456 PMC5404842

[B74] Leyvand T. Cohen-Or D. Dror G. Lischinski D. (2008). Data-driven enhancement of facial attractiveness. ACM Trans. Graph. 27:38 10.1145/1360612.1360637

[B75] Li M. Cole J. B. Manyama M. Larson J. R. Liberton D. K. Riccardi S. L.. (2017). Rapid automated landmarking for morphometric analysis of three-dimensional facial scans. J. Anat. 230, 607–618. 10.1111/joa.12576 28078731 PMC5345630

[B76] Litke N. Droske M. Rumpf M. Schröder P. (2005). “An image processing approach to surface matching,” in Symposium on Geometry Processing, Vol. 255 (Vienna: Citeseer), 207–216.

[B77] Liu F. van der Lijn F. Schurmann C. Zhu G. Chakravarty M. M. Hysi P. G.. (2012). A genome-wide association study identifies five loci influencing facial morphology in Europeans. PLoS Genet. 8:e1002932. 10.1371/journal.pgen.1002932 23028347 PMC3441666

[B78] Loos H. S. Wieczorek D. Würtz R. P. von der Malsburg C. Horsthemke B. (2003). Computer-based recognition of dysmorphic faces. Eur. J. Hum. Genet. 11, 555–560. 10.1038/sj.ejhg.5200997 12891374

[B79] Louis T. A. (1982). Finding the observed information matrix when using the EM algorithm. J. R. Stat. Soc. Ser B 44, 226–233. 10.1111/j.2517-6161.1982.tb01203.x

[B80] Lowe D. G. (2004). Distinctive image features from scale-invariant keypoints. Int. J. Comput. Vis. 60, 91–110. 10.1023/B:VISI.0000029664.99615.94

[B81] Lu K. H. (1965). Harmonic analysis of the human face. Biometrics 21, 491–505. 14338681

[B82] Lynch J. (2018). Face Off: Law Enforcement Use of Face Recognition Technology. Available online at: https://www.eff.org/wp/law-enforcement-use-face-recognition

[B83] McInnes L. Healy J. (2018). Umap: Uniform manifold approximation and projection for dimension reduction. arXiv [preprint]. arXiv:1802.03426.

[B84] Milborrow S. Bishop T. Nicolls F. (2013). “Multiview active shape models with sift descriptors for the 300-w face landmark challenge,” in Proceedings of the IEEE International Conference on Computer Vision Workshops (Sydney, NSW), 378–385.

[B85] Milborrow S. Nicolls F. (2008). “Locating facial features with an extended active shape model,” in European Conference on Computer Vision (Marseille: Springer), 504–513.

[B86] Milborrow S. Nicolls F. (2014). Active shape models with SIFT descriptors and MARS. VISAPP 1:5 10.1109/ICCVW.2013.57

[B87] Molinaro A. M. Simon R. Pfeiffer R. M. (2005). Prediction error estimation: a comparison of resampling methods. Bioinformatics 21, 3301–3307. 10.1093/bioinformatics/bti499 15905277

[B88] Montúfar J. Romero M. Scougall-Vilchis R. J. (2018). Automatic 3-dimensional cephalometric landmarking based on active shape models in related projections. Am. J. Orthod. Dentof. Orthop. 153, 449–458. 10.1016/j.ajodo.2017.06.028 29501121

[B89] Nakata M. Yu P.-L. Nance W. E. (1976). On facial similarity in relatives. Hum. Biol. 48, 611–621. 976979

[B90] Oh Y.-H. See J. Le Ngo A. C. Phan R. C.-W. Baskaran V. M. (2018). A survey of automatic facial micro-expression analysis: databases, methods, and challenges. Front. Psychol. 9:1128. 10.3389/fpsyg.2018.01128 30042706 PMC6049018

[B91] Orchard T. Woodbury M. A. (1972). A Missing Information Principle: Theory and Applications. Technical Report, Duke University Medical Center, Durham, NC.

[B92] Ott J. Rabinowitz D. (1999). A principal-components approach based on heritability for combining phenotype information. Hum. Hered. 49, 106–111. 10.1159/000022854 10077732

[B93] Oualkacha K. Labbe A. Ciampi A. Roy M.-A. Maziade M. (2012). Principal components of heritability for high dimension quantitative traits and general pedigrees. Stat. Appl. Genet. Mol. Biol. 11, 1544–6115. 10.2202/1544-6115.1711 22499698

[B94] Ozcan A. McLeod E. (2016). Lensless imaging and sensing. Annu. Rev. Biomed. Eng. 18, 77–102. 10.1146/annurev-bioeng-092515-010849 27420569

[B95] Papageorgiou C. P. Oren M. Poggio T. (1998). “A general framework for object detection,” in Sixth International Conference on Computer Vision (Bombay), 555–562.

[B96] Paternoster L. Zhurov A. I. Toma A. M. Kemp J. P. Pourcain B. S. Timpson N. J.. (2012). Genome-wide association study of three-dimensional facial morphology identifies a variant in pax3 associated with nasion position. Am. J. Hum. Genet. 90, 478–485. 10.1016/j.ajhg.2011.12.021 22341974 PMC3309180

[B97] Peng S. Tan J. Hu S. Zhou H. Guo J. Jin L.. (2013). Detecting genetic association of common human facial morphological variation using high density 3d image registration. PLoS Comput. Biol. 9:e1003375. 10.1371/journal.pcbi.1003375 24339768 PMC3854494

[B98] Petrie A. Sabin C. (2013). Medical Statistics at a Glance. Chichester: John Wiley & Sons.

[B99] Pritchard J. K. Przeworski M. (2001). Linkage disequilibrium in humans: models and data. Am. J. Hum. Genet. 69, 1–14. 10.1086/321275 11410837 PMC1226024

[B100] Ranjan R. Patel V. M. Chellappa R. (2017). Hyperface: a deep multi-task learning framework for face detection, landmark localization, pose estimation, and gender recognition. IEEE Trans. Pattern Anal. Mach. Intell. 41, 121–135. 10.1109/TPAMI.2017.2781233 29990235

[B101] Richmond S. Howe L. J. Lewis S. Stergiakouli E. Zhurov A. (2018). Facial genetics: a brief overview. Front. Genet. 9:462. 10.3389/fgene.2018.00462 30386375 PMC6198798

[B102] Rolfe S. Lee S.-I. Shapiro L. (2018). Associations between genetic data and quantitative assessment of normal facial asymmetry. Front. Genet. 9:659. 10.3389/fgene.2018.00659 30631343 PMC6315129

[B103] Roweis S. T. Saul L. K. (2000). Nonlinear dimensionality reduction by locally linear embedding. Science 290, 2323–2326. 10.1126/science.290.5500.2323 11125150

[B104] Samal A. Iyengar P. A. (1992). Automatic recognition and analysis of human faces and facial expressions: a survey. Pattern Recogn. 25, 65–77. 10.1016/0031-3203(92)90007-6

[B105] Scheib J. E. Gangestad S. W. Thornhill R. (1999). Facial attractiveness, symmetry and cues of good genes. Proc. R. Soc. Lond. B Biol. Sci. 266, 1913–1917. 10.1098/rspb.1999.0866 10535106 PMC1690211

[B106] Schroff F. Kalenichenko D. Philbin J. (2015). FaceNet: a unified embedding for face recognition and clustering. arXiv:1503.03832 [cs] 815–823. 10.1109/CVPR.2015.7298682

[B107] Shaffer J. R. Orlova E. Lee M. K. Leslie E. J. Raffensperger Z. D. Heike C. L.. (2016). Genome-wide association study reveals multiple loci influencing normal human facial morphology. PLoS Genet. 12:e1006149. 10.1371/journal.pgen.1006149 27560520 PMC4999139

[B108] Short L. J. Khambay B. Ayoub A. Erolin C. Rynn C. Wilkinson C. (2014). Validation of a computer modelled forensic facial reconstruction technique using CT data from live subjects: a pilot study. Forens. Sci. Int. 237, 147.e1–147.e8. 10.1016/j.forsciint.2013.12.042 24529418

[B109] Spreeuwers L. (2011). Fast and accurate 3d face recognition using registration to an intrinsic coordinate system and fusion of multiple region. Proc. Int. J. Comput. Vis. 93, 389–414. 10.1007/s11263-011-0426-2

[B110] Stenton D. R. Keenleyside A. Trepkov D. P. Park R. W. (2016). Faces from the franklin expedition? Craniofacial reconstructions of two members of the 1845 northwest passage expedition. Polar Rec. 52, 76–81. 10.1017/S0032247415000248

[B111] Storms A.-S. Vansant L. Shaheen E. Coucke W. de Llano-Pérula M. C. Jacobs R.. (2017). Three-dimensional aesthetic assessment of class ii patients before and after orthognathic surgery and its association with quantitative surgical changes. Int. J. Oral Maxillof. Surg. 46, 1664–1671. 10.1016/j.ijom.2017.07.002 28751183

[B112] Sun Y. Liang D. Wang X. Tang X. (2015). Deepid3: face recognition with very deep neural networks. arXiv [preprint]. arXiv:1502.00873. 10.1109/ICPR.2008.4760973

[B113] Sun Y. Yin L. (2008). “Automatic pose estimation of 3d facial models,” in 19th International Conference on Pattern Recognition, ICPR 2008 (Tampa, FL), 1–4.

[B114] Swennen G. R. (2006). “3-d cephalometric soft tissue landmarks,” in Three-Dimensional Cephalometry (New York, NY: Springer), 183–226.

[B115] Szegedy C. Liu W. Jia Y. Sermanet P. Reed S. Anguelov D. (2015). “Going deeper with convolutions,” in Conference on Computer Vision and Pattern Recognition (CVPR) (Boston, MA).

[B116] Taylor H. O. Morrison C. S. Linden O. Phillips B. Chang J. Byrne M. E.. (2014). Quantitative facial asymmetry: using three-dimensional photogrammetry to measure baseline facial surface symmetry. J. Craniof. Surg. 25, 124–128. 10.1097/SCS.0b013e3182a2e99d 24406564

[B117] Tenenbaum J. B. Silva V. d. Langford J. C. (2000). A global geometric framework for nonlinear dimensionality reduction. Science 290, 2319–2323. 10.1126/science.290.5500.2319 11125149

[B118] Thornhill R. Møller A. P. (1997). Developmental stability, disease and medicine. Biol. Rev. 72, 497–548. 9375532 10.1017/s0006323197005082

[B119] Tibshirani R. (1996). Regression shrinkage and selection via the lasso. J. R. Stat. Soc. Ser. B 58, 267–288.

[B120] Tsagkrasoulis D. Hysi P. Spector T. Montana G. (2017). Heritability maps of human face morphology through large-scale automated three-dimensional phenotyping. Sci. Rep. 7:45885. 10.1038/srep45885 28422179 PMC5395823

[B121] Turk G. O'brien J. F. (1999a). “Shape transformation using variational implicit functions,” in Proceedings of the 26th Annual Conference on Computer Graphics and Interactive Techniques (Los Angeles, CA: ACM Press; Addison-Wesley Publishing Co.), 335–342.

[B122] Turk G. O'brien J. F. (1999b). Variational Implicit Surfaces. Technical Report, Georgia Institute of Technology.

[B123] Turk M. Pentland A. (1991). Eigenfaces for recognition. J. Cogn. Neurosci. 3, 71–86. 23964806 10.1162/jocn.1991.3.1.71

[B124] van der Maaten L. Hinton G. (2008). Visualizing data using t-SNE. J. Mach. Learn. Res. 9, 2579–2605.

[B125] Vandenberg S. G. Strandskov H. H. (1964). A comparison of identical and fraternal twins on some anthropometric measures. Hum. Biol. 36, 45–52. 14122589

[B126] Vidal R. Ma Y. Sastry S. S. (2016). Generalized Principal Component Analysis, Vol. 5. New York, NY: Springer. 10.1109/TPAMI.2005.244 16355661

[B127] Viola P. Jones M. (2004). Robust real-time face detection. Int. J. Computer Vision. 57, 137–154.

[B128] Vollmar T. Maus B. Wurtz R. P. Gillessen-Kaesbach G. Horsthemke B. Wieczorek D.. (2008). Impact of geometry and viewing angle on classification accuracy of 2d based analysis of dysmorphic faces. Eur. J. Med. Genet. 51, 44–53. 10.1016/j.ejmg.2007.10.002 18054308

[B129] Wallace G. K. (1991). The JPEG still picture compression standard. Commun. ACM 34, 30–44. 10.1145/103085.103089

[B130] Wang Y. Fang Y. Jin M. (2007). A ridge penalized principal-components approach based on heritability for high-dimensional data. Hum. Hered. 64, 182–191. 10.1159/000102991 17536212

[B131] Wilamowska K. Wu J. Heike C. Shapiro L. (2012). Shape-based classification of 3d facial data to support 22q11.2ds craniofacial research. J. Digit. Imaging 25, 400–408. 10.1007/s10278-011-9430-x 22086243 PMC3348987

[B132] Winter R. M. (1996). What's in a face? Nat. Genet. 12, 124–129. 8563748 10.1038/ng0296-124

[B133] Wiskott L. Fellous J.-M. Kuiger N. Von Der Malsburg C. (1997). Face recognition by elastic bunch graph matching. IEEE Trans. Pattern Anal. Mach. Intell. 19, 775–779.

[B134] Wiskott L. Von Der Malsburg C. (1996). Recognizing faces by dynamic link matching. Neuroimage 4, S14–S18. 10.1006/nimg.1996.0043 9345518

[B135] Witten D. M. Tibshirani R. Hastie T. (2009). A penalized matrix decomposition, with applications to sparse principal components and canonical correlation analysis. Biostatistics 10, 515–534. 10.1093/biostatistics/kxp008 19377034 PMC2697346

[B136] Yang M.-H. Kriegman D. J. Ahuja N. (2002). Detecting faces in images: a survey. IEEE Trans. Pattern Anal. Mach. Intell. 24, 34–58. 10.1109/34.982883

[B137] Zadeh A. Lim Y. C. Baltrušaitis T. Morency L.-P. (2017). “Convolutional experts constrained local model for 3d facial landmark detection,” in Proceedings of the IEEE International Conference on Computer Vision Workshops, Vol. 7 (Venice).

[B138] Zhang J. Shan S. Kan M. Chen X. (2014). “Coarse-to-fine auto-encoder networks (cfan) for real-time face alignment,” in European Conference on Computer Vision (Zurich: Springer), 1–16.

[B139] Zhang Z. Luo P. Loy C. C. Tang X. (2016). Learning deep representation for face alignment with auxiliary attributes. IEEE Trans. Pattern Anal. Mach. Intell. 38, 918–930. 10.1109/TPAMI.2015.2469286 27046839

[B140] Zheng Y. Liu D. Georgescu B. Nguyen H. Comaniciu D. (2015). “3d deep learning for efficient and robust landmark detection in volumetric data,” in Medical Image Computing and Computer-Assisted Intervention – MICCAI 2015, Lecture Notes in Computer Science (Cham: Springer), 565–572.

[B141] Zhou D. Petrovska-Delacretaz D. Dorizzi B. (2009). “Automatic landmark location with a combined active shape model,” in Proceedings of the 3rd IEEE International Conference on Biometrics: Theory, Applications and Systems, BTAS'09 (Piscataway, NJ: IEEE Press), 49–55.

[B142] Zou H. Hastie T. Tibshirani R. (2006). Sparse principal component analysis. J. Comput. Graph. Stat. 15, 265–286. 10.1198/106186006X113430

